# Occupational Air Pollution and Cognitive Health: A Systematic Review and Meta‐analysis

**DOI:** 10.1002/alz70860_104170

**Published:** 2025-12-23

**Authors:** Suvarna Jyothi Kantipudi

**Affiliations:** ^1^ Department of Psychiatry, SRIHER, Chennai, Tamilnadu, India

## Abstract

**Background:**

Occupational exposure to airborne pollutants, including particulate matter (PM2.5, PM10), volatile organic compounds, and black carbon, poses significant health risks. Emerging evidence suggests a potential link between such exposures and adverse cognitive outcomes, including neurodegenerative disorders like Alzheimer's disease and Parkinson's disease. This systematic review aims to synthesize findings on the impact of occupational air pollution exposure on cognitive health, providing insights into its role in cognitive decline and impairment.

**Method:**

A systematic search will be conducted across multiple databases, including PubMed, Web of Science, and Scopus, focusing on observational studies, cohort studies, and case‐control designs. Eligible studies will compare workers in high‐exposure occupational environments to those with minimal exposure or baseline cognitive health assessments in longitudinal studies. Inclusion criteria will be studies with standardized cognitive health outcomes, while studies on general ambient air pollution or unrelated chemical exposures will be excluded. Data extraction and quality appraisal will be performed independently by two reviewers, and meta‐analyses will be conducted where possible.

**Result:**

Preliminary analysis indicates a growing body of evidence linking occupational air pollution exposure with adverse cognitive outcomes. Initial trends suggest that workers exposed to high levels of particulate matter and black carbon may exhibit greater cognitive decline and increased risk of neurodegenerative diseases compared to low‐exposure groups. Data synthesis and quality assessment are ongoing to confirm these findings and determine effect sizes.

**Conclusion:**

Though data analysis is still underway, emerging patterns highlight the potential cognitive risks associated with occupational air pollution. These findings emphasize the need for targeted workplace interventions and policies to protect cognitive health among vulnerable occupational groups.

